# Supplementary material for: Identification of phenotypically, functionally, and anatomically distinct stromal niche populations in human bone marrow based on single-cell RNA sequencing
Source: eLife. 2023 Mar 6;12:e81656. doi: 10.7554/eLife.81656 (PMC10097421; doi:10.7554/eLife.81656)
Supplement: Supplementary file 1. [file elife-81656-supp1.docx]

Supplementary File 1. Donor information

| Sample ID | Age | Gender | Cell population |
| --- | --- | --- | --- |
| BM_Y1 | 19 | F | CD45-CD235a- |
| BM_Y2 | 22 | M | CD45-CD235a- |
| BM_O1 | 52 | M | CD45-CD235a- |
| BM_O2 | 53 | F | CD45-CD235a- |
| CD271_Y3 | 21 | F | CD45-CD235a-CD271+ |
| CD271_Y4 | 25 | M | CD45-CD235a-CD271+ |
| CD271_Y5 | 32 | M | CD45-CD235a-CD271+ |
| CD271_O3 | 58 | F | CD45-CD235a-CD271+ |
| CD271_O4 | 61 | M | CD45-CD235a-CD271+ |
