# Supplementary material for: Identification of phenotypically, functionally, and anatomically distinct stromal niche populations in human bone marrow based on single-cell RNA sequencing
Source: eLife. 2023 Mar 6;12:e81656. doi: 10.7554/eLife.81656 (PMC10097421; doi:10.7554/eLife.81656)
Supplement: Supplementary file 2. [file elife-81656-supp2.docx]

Supplementary File 2. Cluster annotation

| Cluster ID | Annotation | Marker(s) |
| --- | --- | --- |
| 0 | Basal cell-like | KRT5 |
| 1 | B-cell progenitors | DNTT, VPREB1, VPREB3, CD79A, CD79B, IGLL1 |
| 2 | B-cell progenitors | DNTT, VPREB1, VPREB3, CD79A, CD79B, IGLL1 |
| 3 | Stromal cells | CXCL12, VCAN, LEPR |
| 4 | Plasma cells | IGHA1, IGHA2, IGKC |
| 5 | Stromal cells | CXCL12, VCAN, LEPR |
| 6 | Stromal cells | CXCL12, VCAN, LEPR |
| 7 | HSPC/CD34-enriched | CD34, PROM1 (CD133), CRHBP, AVP, MLLT3, FAM30A, GATA1 |
| 8 | Stromal cells | CXCL12, VCAN, LEPR |
| 9 | Megakaryocytes | PF4, GP9, PPBP, PPBPP2 |
| 10 | Dendritic cells | FLT3, PLAC8, PLD4, GZMB, IRF7, IRF8, NAPSB |
| 11 | T cells | IL7R, CD3D, CD3E, CD3G |
| 12 | Plasma cells | IGKC, IGHA1, IGHA2, IGLC3 |
| 13 | Plasma cells | IGKC, IGHG1, IGHG2, IGHG3, IGHG4, IGHGP |
| 14 | Granulocytic cells | DEFA3, DEFA4 |
| 15 | Erythroid cells | HBB, HBA1, HBA2, AHSP, TFRC |
| 16 | Stromal cells | CXCL12, VCAN, LEPR |
| 17 | NK cells | NCAM1, GZMH, GNLY, GZMA, IL32 |
| 18 | Granulocytic cells | MPO, SRGN |
| 19 | Granulocytic cells | MPO, AZU1, PRTN3, SRGN, ELANE |
| 20 | Dendritic cells | PLAC8, GZMB, IRF8, PLD4 |
| 21 | Dendritic cells | PLAC8, STMN1, IRF8 |
| 22 | Plasma cells | IGLC2, IGHG1, IGHG3, IGLC3, IGHGP, IGKC |
| 23 | Stromal cells | CXCL12, VCAN, LEPR |
| 24 | Dendritic cells | SCT, CST3, PLAC8 |
| 25 | Plasma cells | IGHG1, IGHGP, IGHG3, IGKC, IGHG4 |
| 26 | Granulocytic cells | CLC, SRGN, MS4A3, ANXA1 |
| 27 | Erythroid cells | HBB, HBA1, HBA2, AHSP, TFRC |
| 28 | Endothelial cells | PECAM1, ICAM2 |
| 29 | Stromal cells | CXCL12, VCAN, LEPR |
| 30 | Plasma cells | IGKC, IGHG3, IGHG1 |
| 31 | Granulocytic cells | AZU1, ELANE, MPO, SRGN |
| 32 | B-cell progenitors | DNTT, VPREB1, VPREB3, CD79A, CD79B, IGLL1 |
| 33 | Granulocytic cells | CEACAM8, PGLYRP1, TCN1 |
| 34 | B-cell progenitors | CD79A, CD79B |
| 35 | NK cells | NCAM1, GZMH, GNLY, GZMA, GZMB, IL32 |
| 36 | Monocytes | CSF1R, CD14, CD33, ITGAM (CD11B), CD86 |
| 37 | Stromal cells | CXCL12, VCAN, LEPR |
| 38 | Stromal cells | CXCL12, VCAN, LEPR |
| 39 | Neuronal cell-containing | NEUROD1, CHGB, ELAVL4, STMN2, INSM1 |
| 40 | Dendritic cells | NAPSB, FLT3, IRF8, PLD4, IRF7 |
| 41 | Erythroid cells | HBB, HBA1, HBA2, AHSP, TFRC |

Abbreviations: HSPC, hematopoietic stem/progenitor cells; NK, natural killer
