# Supplementary material for: Identification of phenotypically, functionally, and anatomically distinct stromal niche populations in human bone marrow based on single-cell RNA sequencing
Source: eLife. 2023 Mar 6;12:e81656. doi: 10.7554/eLife.81656 (PMC10097421; doi:10.7554/eLife.81656)
Supplement: Supplementary file 7. [file elife-81656-supp7.docx]

Supplementary File 7. Characterization summary of stromal clusters.

| Cluster ID | Group ID | Annotation |  | | | | Characterizations | | |
| --- | --- | --- | --- | --- | --- | --- | --- | --- | --- |
|  |  |  | CFU-F | In vitro differentiation capacity | | | Surface phenotype | Localization | Top-10 expressed genes |
|  |  |  |  | Osteo- | Adipo- | Chondro- |  |  |  |
| 3 | A | MSSC | +++ | + | + | + | CD45^-^CD235a^-^CD71^-^CD271^+^CD52^-^NCAM1^-^**CD81^++^** | Peri-vascular, peri-adipocytic and endosteal regions | CXCL12, CFD, IFTM3, IGFBP7, APOE, GAS6, B2M, MDK, PTGDS, DCN |
| 5 |  | HAGEP | ++ | + | + | (+) | CD45^-^CD235a^-^CD71^-^CD271^+^CD52^-^NCAM1^-^**CD81^+^** |  | SOCS3, FOS, FOSB, JUNB, EGR1, ZFP36, IFITM3, NNMT, APOE, CFD |
| 16 |  | Balanced prog. | +- |  |  |  | CD45^-^CD235a^-^CD71^-^CD271^+^CD52^-^NCAM1^-^**CD81^+-^** |  | CXCL12, APOE, CFD, LEPR, IFITM3, CHL1, CP, VCAN, DCN, PTGDS |
| 38 |  | Pre-osteoblast | +- |  |  |  | CD45^-^CD235a^-^CD71^-^CD271^+^CD52^-^NCAM1^-^**CD81^-^** |  | MALAT1, AC010970.1, AD000090.1, FOS, ZFP36L1, FOSB, EGR1, CXCL12, VCAN, CP |
| 29 | B | OC | + | ++ | - | ++ | CD45^-^CD235a^-^CD71^-^CD271^+^CD52^-^**NCAM1^+^** | Endosteal bone-lining areas | LUM, DCN, FOS, TPM1, CLEC11A, S100A13, VIM, IBSP, PCOLCE, COL1A2 |
| 23 |  |  |  |  |  |  |  |  | S100A13, EEF1A1, TPM1, TPT1, AC010970.1, ADIRF, FTH1, BGN, SPP1, AD000090.1 |
| 6 | C | Pre-fibroblast | +- | + | - | +- | CD45^-^CD235a^-^CD71^-^CD271^+^**CD52^+^**NCAM1^-^ |  | CXCL12, APOE, CFD, IFITM3, PTGDS, IGFBP2, LEPR, NNMT, FOS, SOCS3 |
| 37 |  |  |  |  |  |  |  |  | PRTN3, AZU1, MPO, SRGN, ELANE, CTSG, APOE, GSTP1,CXCL12, CFD |
| 8 |  |  |  |  |  |  |  |  | S100A9, APOE, S100A8, CXCL12, CFD, IFITM3, LEPR, VCAN, FOS, PTGDS |
